# Oral Histories, Public Engagement, and the Making of *Positive in Prison*

**DOI:** 10.1093/hwj/dbz009

**Published:** 2019-02-21

**Authors:** Janet Weston

## Abstract

How might creative practices surrounding oral history contribute to public engagement and to historical research itself? These questions are considered here through a reflective account of the making of the audio drama *Positive in Prison: HIV Stories from a Dublin Jail*. *Positive in Prison* is based on oral histories of HIV/AIDS in the Republic of Ireland, gathered in 2016–17 as part of the Wellcome Trust-funded project ‘Prisons, Medical Care and Entitlement to Health in England and Ireland, 1850–2000’. This piece reviews the processes and practical considerations behind the making of the audio drama and its associated launch events, alongside a summary of the history of HIV/AIDS and of prisons that was being produced and shared. It also offers reflections on the advantages and disadvantages of this particular project in relation to public engagement, the uses of oral histories, and creative history-telling.

It may not always be acknowledged, but imagination and creativity are at the heart of most, if not all, of the activities labelled as ‘history’. Innovative practices around oral history and public engagement offer opportunities for embracing and thinking critically about this aspect of history work, and for learning and enjoyment on the part of historians as well as their interviewees, publics, and professional partners. Such practices can prompt valuable reflection on the uses of oral histories, on the position and purpose of collaboration in historical research, and more fundamentally, on how and why we all create and present stories about the past.

In late 2016, the team behind the research project ‘Prisons, Medical Care and Entitlement to Health in England and Ireland, 1850–2000’, funded by the Wellcome Trust,^1^ applied for additional funding to develop public engagement. One of our proposed activities was to create an audio drama based on oral history interviews, gathered for the strand of the project which examined the impact of HIV/AIDS on prisons. This application was successful and we began work on the audio drama in April 2017. The process of creating this drama and its launch events presented unexpected hurdles and rewards, and showed that good public engagement, like good historical analysis, requires us to be critical, careful, and thoughtful. The final product, *Positive in Prison: HIV stories from a Dublin Jail*, was launched in London in November and then Dublin on World AIDS Day, 1 December 2017.^2^

In this article I offer an account of the making of *Positive in Prison*. The first two sections combine an overview of the processes and practical considerations from inception to delivery, with a summary of the history that was being produced and shared. In the final section, I reflect upon the advantages and disadvantages of this particular project as it evolved, and how I might approach it differently a second time around. In so doing, I hope to add to debate about public engagement, representations and uses of oral histories, and creative history-telling, but most of all, I hope to encourage historians to think imaginatively, if carefully, about new ways of doing history.

## Grand Designs

When the prospect of further funding for public engagement emerged, I was in the midst of conducting oral history interviews as part of my research into the impact of HIV/AIDS on healthcare for prisoners in England and Ireland.^3^ Many of these interviews had been enormously compelling, with addiction workers, probation officers, and medical professionals sharing moving and sometimes astonishing memories of HIV/AIDS as it began to affect prisons in the 1980s and 1990s. These were an obvious starting point for some form of drama or performance. With assistance from the London School of Hygiene & Tropical Medicine’s (LSHTM) Public Engagement Officer Rebecca Tremain and her contacts, we prepared a brief outline and all-important budget. The proposal was to ‘develop oral histories around HIV/AIDS into a script with rehearsed readings in London and Dublin’. Recordings were to be ‘hosted on the project website and other platforms worldwide’, with the potential for touring live performances at a later date.

News that the funding had been awarded arrived in April, and prompted several exciting meetings with our collaborators, Kate Valentine and Alison Ramsey of the production company Digital Drama.^4^ Kate and Alison had experience of working with academic institutions, including LSHTM, to create historically engaged public events and performances, and they brought a combination of imagination and practical know-how. They immediately looked out for a writer to help transform hours of interviews and pages of archival documents into a coherent script. We also began scouting for venues for the rehearsed readings, and quickly booked the LSHTM’s small recording studio for a day in November to turn the script into an audio drama. Digital Drama set out a provisional timetable, ending with rehearsed readings in Dublin in September and London in December on World AIDS Day, 1 December, seven months in the future and one month before the end of my contract.

Long conversations with Digital Drama about the research project and its themes were essential, and began to suggest that the oral history interviews focusing on Ireland, rather than England, might offer the best material. There were two reasons for this. Firstly, the larger prison estate of England & Wales had proved harder for me to capture through interviews. Over the last few decades of the twentieth century, England & Wales was home to nearly 100 prisons, tens of thousands of prison staff, including many based in the Prison Department of the Home Office in London, and between 40,000 and 65,000 inmates at any given moment. In the Republic of Ireland, there were just ten prisons in the early 1980s and only a few more by the end of the century. Prisoner numbers were increasing, but did not exceed 4,000 until the 2000s. Prisons were managed by governors on the ground and a handful of civil servants in the Department of Justice. It was therefore a much smaller and more streamlined service, and my fifteen oral history interviewees had been able to portray it more fully.

Secondly, accounts of the early days of HIV/AIDS in Dublin and of a separate unit for prisoners with HIV/AIDS within Dublin’s Mountjoy prison complex had been particularly compelling. It was a new story, both to my ears and for those telling it, who – with one exception – had not had cause to reflect back on their experiences there before.^5^ A majority of interviewees volunteered information on the same specific subjects from different perspectives, demonstrating the potential for oral history to recreate multiple standpoints and opinions on past events^6^ and suggesting that this could be a valuable feature of our script. These accounts also introduced some of the distinctive features of the Irish response to HIV/AIDS, which remain little-known despite growing interest in histories of HIV/AIDS more broadly.^7^ Lastly, the physical segregation of prisoners with HIV/AIDS, and the small numbers of prisoners and staff thrown together in this separation unit, seemed advantageous for the purposes of telling a story quickly: the running time of our script could not exceed one hour, to avoid stretching both budgets and audience attention spans.

Our first minor stumbling block came with the need to share recorded interviews. To comply with institutional requirements my recordings were encrypted, which eliminated any straightforward sharing options. The only solution we could concoct was to share a small number of encrypted recordings on a memory stick, and for collaborators then to download the same encryption software to access them. Encrypted audio files of full-length interviews were too large, so I selected a handful of clips of around ten minutes each. Providing other primary and secondary sources including password-protected interview notes was far easier, but this initial difficulty meant that the short extracts were the only audio files shared and we relied heavily on written transcripts and notes. Here was a situation with precedent: oral historians have often been accused of venerating the written transcript over the voice of the interviewee.^8^ As Canadian oral historian Stacey Zembrzycki has argued, the accessibility of digital technologies opens up enormous possibilities for sharing audio and even video recordings of oral histories,^9^ but the question of ethical and secure methods of sharing still requires consideration.

In June 2017, a hasty application for one of our rehearsed readings to be part of the *Being Human* Festival of the Humanities later that year^10^ meant that the audio drama had to be given a name, and *Positive in Prison* was born. That same month, I had a lengthy conversation with the writer recruited to the project. She had listened to and read the material provided, and had many insightful questions. We talked about the impact of Dublin’s heroin epidemic, which had begun to take hold in around 1979 and shaped the spread and perception of HIV/AIDS as well as the characteristics of the prison population. We talked about the criminalization (until 1993) of homosexuality in the Republic of Ireland, the role and views of the Governor of Mountjoy prison during the 1980s and 1990s, and the impact of HIV/AIDS upon those working in the fields of addiction and probation. We also talked about the emotions expressed in some of my interviews and in sources from the 1980s, particularly an episode from January 1986 of *Today Tonight*, the leading current affairs programme on Irish television. In this we could hear from the first prisoner in Ireland to be diagnosed with HIV and from the brother of another inmate who had taken his own life at around the same time.^11^ We also discussed those overarching questions that are key for historians as for storytellers of other kinds: why did events unfold in this way, and not in another? What was it like for those involved? We wondered why, exactly, the Irish prison service had set up a separate unit for prisoners with HIV/AIDS, what that unit had been like for prisoners and staff, and why it survived as long as it did – for nearly ten years.

Happily, these conversations coincided with work on an article about the different policies around HIV/AIDS that were adopted in Ireland and England & Wales,^12^ and ideas flowed between the two projects. Histories of HIV/AIDS have often dwelt upon reactions within medicine, politics and the media which all appeared to embody Foucauldian views of disease, difference, and power. Historical work on HIV/AIDS policy in the late 1980s and 1990s, taking a slightly different tack, emphasized the influence of expert groups, including a new constituency of experts from the gay community.^13^ The story emerging from this work on prisons suggested that reactions were still more complex, with responses to HIV/AIDS deeply influenced by pre-existing institutional structures and preoccupations, in combination with the nature and extent of external pressures and scrutiny. The history of HIV/AIDS in Mountjoy prison exemplified this, and opened up new ways of looking at the recent history of prisons and the history of HIV/AIDS in Ireland. Periodization can be difficult for recent history, but the first diagnosis of ‘AIDS’ in an Irish prison and the panic that ensued was a pivotal moment. This was at the heart of *Positive in Prison* as it began to take shape over the summer of 2017.

## *Positive in Prison* in Development

Acceptance onto the programme for the *Being Human* festival meant that we had to commit to a date in late November for the London event. Our World AIDS Day launch relocated to Dublin, and colleagues at University College Dublin began hunting for a possible venue. This search was hindered by the fact that we still had no clear idea of what the event would be: a rehearsed reading? A full performance? How would the pre-recorded audio drama fit in? We had planned to have a panel of expert speakers accompanying the performance of *Positive in Prison*, but how would these elements interact? Such questions were resolved only when the final draft of the script was in hand.

The first draft of the script arrived in September. It was a rich drama, weaving many themes from the oral history interviews into a work of mostly fiction. The first diagnosis of HIV amongst Mountjoy’s inmates was its starting point, and it showed staff, prisoners, and their families all struggling with fear and misinformation. Over a slightly condensed timeline, the script conveyed the anxieties of prisoners about their own health and the concerns of staff and management that AIDS, or fear of AIDS, could prompt serious disruption. True to the events of 1986, it showed prisoners diagnosed with HIV being moved to another prison nearby, Arbour Hill, where sex offenders were typically housed, and the rooftop riots that ensued. To regain control, the Department of Justice determined that the Mountjoy segregation unit, previously used for political prisoners being moved around the prison estate, would become home for this new cohort of troublesome inmates. After touching on the mixed reactions of medical professionals, addiction workers and prison staff to those in the separation unit, most of whom were young heroin users, the draft script ended with the death of a prisoner from AIDS-related illness in the hospital opposite Mountjoy, an event which had been described to me in an earlier interview.

I reviewed this draft with particular attention to details that seemed inaccurate or lines that felt anachronistic, while Digital Drama reviewed it from their creative and practical perspective. It was so promising that we began to talk about finding a wider audience, perhaps through radio, and Digital Drama made contact with producers in Dublin. This was where we met our second, more significant, stumbling block. Their reaction to the draft script was profoundly negative. It was perceived as unduly critical of the Irish prison system and of reactions to HIV/AIDS, and insensitive in its approach; the fact that Digital Drama, the writer and I were all English did not go unnoticed. This was a salient reminder of some of the challenges that public engagement brings. Historical research can address contemporary themes and contribute to current debate, but this very vitality may prompt strong reactions. This is not to criticize our critics: although I was impressed by the first draft, with hindsight its dramatic and semi-fictionalized rendering may not have been right for our story. It had largely removed the interviewees themselves and the underlying research processes, and risked a (melo)dramatic presentation which blurred fact and fiction in an unhelpful way. As we reconsidered our approach, the process of rethinking and redrafting anchored events more firmly in their specific time and place, and made much greater use of the words of my interviewees, bringing their powerful accounts to the fore. This became a significant strength of the finished piece.

Time and deadlines were pressing. We agreed to a rewrite, using the voice of a narrator to introduce the bigger picture of HIV/AIDS in the 1980s before focusing on the situation at Mountjoy prison. This overarching narrative would be supported by verbatim extracts from the oral histories, grounding the piece in the views and experiences of those who were there. Our writer, already steeped in the material, prepared the narrative and suggested extracts to use. The autumn passed in a blur of script revisions.

This new approach created a much more overtly factual piece, documentary in style, still focusing upon the HIV/AIDS separation unit but contextualizing it much more clearly. The oral histories had greater prominence but were framed by explanatory statements to help give the piece shape and meaning. Typically, oral histories presented in this way might include an introduction or epilogue explaining the interview process and perhaps even describing the relationship between interviewer and subject in detail, as in *The Autobiography of Malcolm X*.^14^ In this case, we chose to include an epilogue, in which the origins of the verbatim extracts and the background to the research project were explained.

The epilogue also affirmed that the words had been voiced by actors. The use of actors and not clips from the original recordings was a practical necessity. My recordings were from different spaces with different qualities of sound, making it hard to knit together the original extracts. I had also conducted two ‘group’ interviews, which presented further obstacles to the use of original clips, given the overlapping voices and even lower sound quality. Furthermore, a few interviewees had requested anonymity, meaning that we could not use these original recordings in any case. The use of actors brought advantages, too: it connected the piece to dramatic traditions as well as those of documentary, even with the use of verbatim extracts, hinting at the interpretative and creative processes underlying the piece and, by extension, the historical research behind it.

The revised script emerged swiftly and required relatively little editing from me, although the process of weaving together verbatim extracts was onerous for the writer and for Digital Drama: hundreds of extracts on slips of paper were physically moved around an outline of the script’s structure to create a coherent (and sufficiently short) piece. This iteration of the story began with the emergence of a heroin epidemic in Dublin, and moved from there to the arrival of HIV/AIDS amongst injecting drug users, and to the first diagnoses in prison. Emphasizing the lack of knowledge and planning around HIV/AIDS, in both individual and structural terms, it explained the panic and fear surrounding diagnoses in prison, and placed the creation of the separation unit in that context. My interviewees had not held back in their criticism of the unit, and the script included references to poor conditions, negative impacts upon mental health, and concealment of HIV diagnoses amongst other prisoners, to mention a few. The enormous impact of AIDS on some families and areas of Dublin also came across strongly. It also featured more positive memories of the unit, of a sense of community and of staff doing their best in difficult circumstances, and described some of the resistance to the closure of the unit.

This more documentary model encouraged us to commit to launch events that followed a fairly traditional academic pattern. A live performance of this script was not viable, and we decided to begin the launch events by dimming the lights and playing out the recorded docudrama in full. This experiment in communal listening would be followed by a panel discussion, chaired by the *Positive in Prison* narrator, and we sought out experts on contemporary public health to participate and complement our historical expertise. Holding the Dublin event on World AIDS Day meant that some potential guests and speakers were already committed elsewhere, not least because of a celebration of the thirtieth anniversary of the organization *HIV Ireland* that same evening, hosted by the President of Ireland. The availability and costs of venues at this relatively late stage also presented some problems, but we were able to use a lecture theatre at the LSHTM in London for no charge, and in Dublin we booked space at the home of the Royal College of Physicians of Ireland, a beautiful building in the centre of the city. A good quality recording of the docudrama itself was essential, and required professional actors, sound engineering, directing and editing. Here, Digital Drama’s expertise was invaluable. Sitting in on an initial read-through and some of the recording itself revealed to the uninitiated just how much work lay behind the capture of every line, and the final piece was given depth and a professional quality with further editing afterwards. By mid November, *Positive in Prison* was ready to be heard.

## Reflections

What does this experience offer to historians and others interested in public engagement, impact, oral histories and/or creativity in historical research? Did this project fulfil the ideals of oral history and public engagement, and did it make good use of the potential in both strands of work for more creative and diverse methods? Beginning with the positive, the use of oral history interviews within audio productions provides one obvious way to preserve some of that which is lost in the more usual process of turning oral history into written material. As Shelley Trower has summarized, oral histories (like any other historical source) demand that choices are made about what to leave out and what to include, but something very particular can be lost when oral histories are expressed and analysed in written form: the gestures, the performance, the tone and emotional content, the exchange between interviewer and interviewee, the voice itself. Scholars have made a case for the importance of these aspects for communities in which written history is outweighed by oral tradition,^15^ and we might add that it can also be important for communities and individuals whose voices are not often heard, sometimes literally. This connects with views of oral history as fundamentally collaborative and empowering, as articulated by Paul Thompson. Ways of reproducing some of these elements of orality have been discussed and attempted in a variety of media,^16^ and an audio docudrama afforded another way of retaining the sound and qualities of spoken interviews.

As a recording rather than a script for live performance, *Positive in Prison* could be made available online without any costs attached to its future use. This allowed all of those involved in the research to hear it, not just those who could attend events. The recording became an important representation of their contributions for those who had helped me throughout my research, few of whom would read and enjoy an academic article. It also allowed the piece to have international and longer-term reach, and to be adopted and used in practical ways: the director of a drug addiction service in Dublin reported that it would be useful for new recruits to the organization, to help them understand their clients’ past experiences and the broader context of attitudes around addiction services and blood-borne viruses. In terms of accessibility, longevity and scope for combining historical sources and analysis with creative structures and presentation, the audio docudrama format may be unusual but has much to recommend it.

Discussions at the launch events were fascinating: the recording provided an excellent starting point for both historical and contemporary reflection. In London, the audience was mostly made up of students and practitioners in the field of HIV/AIDS, public health, or penal policy in the UK, and the conversation naturally turned to the differences in Irish history and the position of prisons within HIV/AIDS policy today. In Dublin by contrast, with interview participants in attendance and a smaller venue, audience members were eager to contribute additional memories and their opinions on the material in the docudrama. This event began to resemble a witness seminar, and if we had anticipated this and briefed the chair accordingly, we could have encouraged more sharing and discussion amongst audience members in both locations. These events were also an opportunity to thank interviewees and others involved, with a drinks reception beforehand. Feedback suggested that they were well received: despite my own hesitations about ‘communal listening’, attendees described the docudrama as ‘moving’ and ‘powerful’ and there was little or no awkwardness about the experience. Attendees also reported that it had prompted new insights and questions. One wrote that ‘I now understand the lack of understanding and lack of logical response’ to HIV/AIDS in the Republic of Ireland; another appreciated the opportunity to reflect back on a challenging time in his professional and personal life.

These responses, not surprisingly, generated considerable satisfaction for the team behind *Positive in Prison*, and added to my enjoyment in working on the whole project. Such enjoyment is an advantage of both public engagement and creative approaches to history which should not be disregarded: scholarly publications and other outputs have their rewards, but this project brought with it a raft of new experiences. It also provided a heightened awareness of the role of storytelling within history, and the opportunity to pay attention to some of the emotions, confusions and fragments of anecdote and evidence that accrue to the research process, but are generally excised from peer reviewed publications. Indeed, the docudrama form, along with many other creative ways of sharing history, can highlight parts of historical work that are often masked. Referring to the use of visual art in public history, Karen Harvey has written that it is capable of ‘recreating what is hidden or absent in the historical record’,^17^ and creative approaches can also draw attention to what remains unknown or uncertain. Perhaps the best example of this was in the first draft of *Positive in Prison*. I had been unable to trace any former prisoners to interview, despite a few promising leads, and the writer marked this absence with striking moments of silence at key points in the script, such as when the prison doctor delivered his first diagnosis of HIV. This was lost in the more documentary final form, but even this format could draw attention to the subjective processes involved in historical analysis by featuring different accounts of the same events. Listeners noticed the sometimes surprising interpretations that individuals put forward in the verbatim extracts, and began to engage in their own analysis of these sources.

The decision to include verbatim extracts was a particular strength of the finished piece. Actor and playwright Robin Soans has reflected that verbatim theatre, in which actors deliver the spoken words of real people, does not so much give an interviewee ‘a voice – he has a voice already – but it does provide his voice with listening ears: mine when he tells me his story, and those of the audience when the actor tells it to them’. It provides ‘an amplification of an otherwise lost voice’.^18^ Through the use of oral histories within the *Positive in Prison*, and the listening experiences at the launch events, we as audience were made more aware of this act of listening and of the potential for these voices to go unheard. Listening, as theatre critics have argued, ‘rarely operates alone: we are usually looking and listening simultaneously, so that even if a particular performance is modelling a new form of listening, it may also perpetuate old spectatorial habits’.^19^ Yet, in the context of an audio docudrama and the unusual experience of listening to this as part of a large group in a lecture theatre, listening could be foregrounded. Particularly with the current popularity of the podcast and the relative ease of creating and sharing digital files, this format offers an accessible way to encourage listening while avoiding these ‘spectatorial habits’, and to ensure that oral histories are heard.

On a more critical note, I wish that we had involved the interviewees to a greater extent in the making of *Positive in Prison*. This would have brought us closer to a form of engagement which values collaboration over dissemination, for which Laura King and Gary Rivett have recently made a persuasive case.^20^ Funders, too, are beginning to emphasize the two-way nature of the engagement they envisage.^21^ A useful model for this can be found in a project to record and represent the memories of the Sailortown Regeneration Group (SRG) in Belfast. Here, artists Fionnuala Fagan and Isobel Anderson collected interviews and then used verbatim extracts to create songs about the area. Importantly, they then entered into ‘a process of sharing and feedback’ with their interviewees, performing the songs for them ‘to clarify they truly represented their stories’. ‘We were aware’, the artists wrote, ‘that, although we had taken the words verbatim from the interviews with the SRG, these songs were inevitably our own artistic interpretation.’ The SRG could share their reactions, and made some requests for changes to the lyrics to add and remove specific names. As Fagan and Anderson explained, this process encouraged interview participants to remain connected to the research going on around them, to find ‘new meaning and value in their memories’, and to retain some control over how their stories would be re-told.^22^

This question of control over material and interpretation is an important consideration for oral history, and is given new complexity and weight by public engagement activities. Such projects can transform and present interviews in unexpected ways. When conducting my interviews, I discussed with participants how recordings would be used before obtaining their consent, but my examples were those familiar to academia: research papers, presentations at conferences and storage in an archive for the benefit of future researchers. The notion of an audio docudrama, freely available online, did not cross our minds. As *Positive in Prison* began to take shape, some participants were concerned, even alarmed, about how their words were being used, who would hear them and what effect this might have on their personal or professional lives. Greater consultation with my interview participants would have been preferable from an ethical point of view and could have benefited the research as well as the docudrama: the scripts and the process of finding a way to tell this story might have prompted additional insights and ideas from interviewees and have encouraged ongoing engagement with the research project. Furthermore, King and Rivett have challenged us to consider whether a failure to value two-way conversations and non-academic expertise within public engagement may ‘reproduce and reinforce intellectual boundaries, and thereby sustain divisions between academics and the public’.^23^ Although we foregrounded the expertise of my interviewees in the docudrama, there was little two-way conversation. I hope that boundaries were not created or reinforced, and I believe that the act of conducting this research and sharing it with a wider audience helped to reduce the gap between research and the community, but the process itself did little to overcome the divisions that King and Rivett describe.

That said, collaboration in the sphere of oral history is not necessarily possible or even desirable. Oral history, like public engagement, may be ‘essentially creative and co-operative’ with potential for co-production,^24^ but how does the co-operative model fare when participants are hostile to each other’s perspectives, or to the historian’s analysis? With elite interviewees or controversial subjects such as HIV/AIDS or penal policy, an ‘official version’ of events might be firmly entrenched and staunchly protected, or efforts to ‘rewrite history’ may still be underway: it is the historian’s role to approach this critically.^25^ Within traditional academic outputs, control over interpretation and analysis tends to remain with the historian for these reasons, and co-production or collaboration is perhaps rare (although historians may seek and incorporate feedback from interviewees more often than is formally acknowledged). Public engagement activities offer a slightly different setting, in which historical analysis is not necessarily front and centre and the expertise of non-historians such as artists, writers, or curators is often vital, but the potential for conflict and the negotiation of boundaries and responsibilities still requires careful consideration.

On a practical level, a greater process of two-way conversation in the process of public engagement would call for a revised budget and revised timetable. Budgets were always tight, not least because our initial costings had not included VAT. This was not identified until a late stage, and here closer liaison from the outset with internal experts in events and administration, contracts and finance would have helped. Our time frame was also stretched to the limit. Ideally, the process of creating our docudrama would have taken place earlier, alongside the oral history interviews and as a part of the research process. This would also permit insights from the process to feed into the ongoing research. After the launch event in Dublin, for example, attendees mentioned friends and acquaintances who had spent time in the Mountjoy separation unit, or in the main men’s prison during the height of fears over AIDS, who could have contributed additional oral histories. But, with little time left on my contract and research publications already submitted for review, these sources came too late.

Timing is a recurring concern. With more time to consider draft scripts, we could have drawn on even more innovative methods of giving narrative meaning to oral history collections, which make more explicit the processes of interpretation. One example can be found in ‘I Can Almost See the Lights of home: a Field Trip to Harlan County Kentucky’. Here, as would be the case with most collections of oral history interviews, the material made little narrative sense without some kind of explanation. The creators were concerned about imposing their own interpretation at the expense of all others, and their solution was to construct a connecting narrative using extracts from a recorded interview between producer Charles Hardy and oral historian, Sandro Portelli.‘What, after all, could make more sense than to interweave the stories of the people contained in Sandro’s interviews with the story of Sandro and me attempting to make sense of those stories?’, Hardy observed. ‘Listeners could now engage with Sandro and me in the search for meaning.’^26^ This kind of creative and imaginative approach could enhance what we do with oral histories, and with our conversations with the public.

Drawing upon Raphael Samuel’s views of public history, Karen Harvey has made a persuasive case for looking ‘past the dominant focus on “learning lessons”, “public debate” and “transferable skills”’ in our conceptualizations of public engagement. As she observes, ‘we come to the world not only through a sceptical assessment of evidence’, but also through emotion and imagination. History with and for the public can provide inspiration for creative thinking on all sides.^27^ Marina Warner has made a similar argument, advocating that historians and other storytellers resist pressure to commit to facts, generated by anxiety about ‘fake news’. ‘Stories’, she writes, ‘build the imponderable but all-important active ingredients of social values, brought about by gradually evolving consensus,’ and the tellers of stories should ‘make the arguments for the truth of the imagination – a romantic mantra, but one that is on the side of life and co-existence and civilisation.’^28^ Both oral history and public engagement demand imagination on the part of all those involved, and offer opportunities to embrace creativity and collaboration in the pursuit of meaningful stories. We should approach these endeavours no less thoughtfully and critically than any other aspect of our researching lives, but as *Positive in Prison* hopefully shows, the approach is one well worth adopting.

## Notes and References

1 For more on this project, see https://histprisonhealth.com/.

2 It can be heard at https://histprisonhealth.com/arts-projects/positive-in-prison-hiv-stories-from-a-dublin-jail/.

3 These interviews are available in the AIDS Archive at the London School of Hygiene and Tropical medicine (LSHTM). We hope that some will also be made available digitally through the University College Dublin Archives.

4 See http://www.digitaldrama.org/.

5 The exception was former Governor of Mountjoy, John Lonergan, who had covered these events in a memoir: John Lonergan, *The Governor*, Dublin, 2010.

6 Paul Thompson, *The Voice of the Past: Oral History* (1978), 3rd edn, Oxford, 2000, p. 6.

7 For examples of this interest see Matt Cook, ‘“Archives of Feeling”: the AIDS Crisis in Britain 1987’, *History Workshop Journal* 83, spring 2017; Victoria A. Harden, *AIDS at 30: a History*, Washington DC, 2012; Richard A. McKay, ‘“Patient Zero”: the Absence of a Patient’s View of the Early North American AIDS Epidemic’, *Bulletin of the History of Medicine* 88, 2014, pp. 161–94; and ‘Epidemic: When Britain Fought AIDS’, Channel 4, 2017. Emerging work on Ireland includes David Kilgannon, ‘How to Survive a Plague: AIDS Activism and Ireland, 1983–9’, unpublished Masters Dissertation, University College Dublin, 2015; Ann Nolan, ‘Marriage Is Not an Anti-Viral Agent’: the Transformation of Sexual Health Policy in the Initial Decade of HIV/AIDS in Ireland’, unpublished PhD thesis, Trinity College Dublin, 2014; Ann Nolan and Fiona Larkan, ‘Vectors of Transnationality in the Adoption of a Liberal Public Health Response to HIV and AIDS in Ireland’, *Global Social Policy* 16: 3, 2016, pp. 253–67.

8 Steven High, ‘Telling Stories: a Reflection on Oral History and New Media’, *Oral History* 38: 1, 2010, pp. 101–12.

9 Stacey Zembrzycki, ‘Bringing Stories to Life: Using New Media to Disseminate and Critically Engage with Oral History Interviews’, *Oral History* 41: 1, 2013, pp. 98–107.

10 A week-long national festival of free public events, mostly with roots in academic research, led by the School of Advanced Study, University of London, in partnership with the Arts & Humanities Research Council and the British Academy. See https://beinghuman-festival.org/, accessed 1 Feb. 2018.

11 Poor quality versions of this programme are currently available on YouTube, for example https://www.youtube.com/watch?v=WFlW1KWPZ6g, accessed 1 Feb. 2018.

12 Janet Weston and Virginia Berridge, ‘AIDS Inside and Out: HIV/AIDS and Penal Policy in Ireland and England & Wales in the 1980s and 1990s’, *Social History of Medicine* 2018, doi:10.1093/shm/hky090.

13 Examples of the former include Simon Watney, *Policing Desire: Pornography, Aids, and the Media*, London, 1987; Dennis Altman, *AIDS and the New Puritanism*, London, 1986. Examples of the latter include Daniel M. Fox, Patricia Day and Rudolf Klein, ‘The Power of Professionalism: Policies for AIDS in Britain, Sweden, and the United States’, *Daedalus* 118, 1989, pp. 93–112; Virginia Berridge, *AIDS in the UK: The Making of Policy, 1981–1994*, Oxford, 1996.

14 Shelley Trower, ‘Auto/Biographical Oral Histories, from “Oral Memoirs” to “The Life of Nate Shaw”’, *Oral History* 45: 1, 2017, pp. 43–54.

15 Trower, ‘Auto/Biographical Oral Histories’.

16 Examples include song, as in Fionnuala Fagan and Isobel Anderson, ‘Stories of the City: an Artistic Representation of Sailortown’s Oral History’, *Oral History* 44: 1, 2016, pp. 108–16; the aural essay, as in Charles Hardy and Alessandro Portelli, ‘I Can Almost See the Lights of Home: a Field Trip to Harlan County Kentucky’, *Journal for MultiMedia History* 2, 1999 (https://www.albany.edu/jmmh/); and forms of poetry, as described over four decades ago in Dennis Tedlock, ‘Learning to Listen: Oral History as Poetry’, *Boundary 2*, 3: 3, 1975, pp. 707–28.

17 Karen Harvey, ‘“Envisioning the Past: Art, Historiography and Public History”’, *Cultural and Social History* 12: 4, 2015, pp. 527–43, p. 536.

18 Quoted in Caroline Wake, ‘Headphone Verbatim Theatre: Methods, Histories, Genres, Theories’, *New Theatre Quarterly* 29, 2013, pp. 321–35, p. 332.

19 Wake, ‘Headphone Verbatim Theatre’.

20 Laura King and Gary Rivett, ‘Engaging People in Making History: Impact, Public Engagement and the World Beyond the Campus’, *History Workshop Journal* 80, 2015, pp. 218–33.

21 The Wellcome Trust, for example, also define public engagement as ‘where researchers share their ideas with the public and in return get new perspectives on their research’: a two-way process. See https://wellcome.ac.uk/funding/research-enrichment-public-engagement, accessed 6 Feb. 2018.

22 Fagan and Anderson, ‘Stories of the City’, p. 10.

23 King and Rivett, ‘Engaging People in Making History’, p. 220.

24 Thompson, *Voice of the Past*, p. 17.

25 These and other issues surrounding oral history are discussed in more detail in Virginia Berridge, ‘Hidden from History? Oral History and the History of Health Policy’, *Oral History* 37, spring 2010, pp. 91–100 and ‘Researching Contemporary History: AIDS’, *History Workshop* 38, 1994, pp. 228–34.

26 ‘Adventures in Sound: Aural History, the Digital Revolution, and the Making of “I Can Almost See the Lights of Home: a Field Trip to Harlan County Kentucky”’, in *Oral History and Digital Humanities: Voice, Access, and Engagement*, ed. Douglas A. Boyd, Mary Larson and Charles Hardy, New York, 2014, pp. 53–75, p. 61.

27 Harvey, ‘Envisioning the Past’, p. 528.

28 Marina Warner, ‘The Truth in Stories’, *History Workshop Online*, 2018: http://www.historyworkshop.org.uk/the-truth-in-stories/, accessed 6 Feb. 2018.

